# Supplementary material for: A natural variation-based screen in mouse cells reveals USF2 as a regulator of the DNA damage response and cellular senescence
Source: G3 (Bethesda). 2023 Apr 25;13(7):jkad091. doi: 10.1093/g3journal/jkad091 (PMC10320765; doi:10.1093/g3journal/jkad091)
Supplement: jkad091_Supplementary_Data [file jkad091_supplementary_data.zip › Supplemental_Methods_G3-2023-404074.docx]

**Primary cell extraction and culture**

Wild-derived, inbred lines of *Mus musculus* (PWK/PhJ), *Mus spretus* (STF/Pas), their interspecies F1 hybrids (*M. musculus* x *M. spretus*), and *M. domesticus* (TUCA, MANB) were maintained in standard conditions under Montana Institutional Animal Care and Use Committee protocol number 062-1JGDBS-120418. Animals were included based on age (3 to 5 months) and availability at the time of request, and were humanely euthanized prior to tissue collection via CO_2_ treatment and cervical dislocation. For each genotype, five tails from two males and three females aged 3 to 5 months were collected into chilled Dulbecco’s Modified Eagle Medium (DMEM) and shipped to the Buck Institute/UC Berkeley for further processing. *M. domesticus* TUCA, from Tucson, Arizona, in their 40th generation of sib-sib mating, and MANB, from Manaus, Brazil, in their 25th generation of sib-sib mating, were maintained in standard conditions under UC Berkeley Institutional Animal Care and Use Committee protocol number AUP-2016-03-8548-2. For each genotype, two tails from female mice less than 10 weeks old were collected as above. No blinding was required for tail collection.

Primary tail fibroblasts were extracted from the cuttings essentially as described (Khan and Gasser 2016). Briefly, tail cuttings first were soaked in 70% ethanol for 5 minutes and then laid out in sterile 10 cm tissue culture dishes in a biosafety cabinet for 5 minutes to dry. The tails were then transferred to a 10-cm dish containing 10 mL of complete medium (RPMI 1640 medium, 10% fetal bovine serum, 50 µM 2-mercaptoethanol, 100 µM asparagine, 2 mM glutamine, and 1% penicillin-streptomycin). Hair was removed from the tails using sterile forceps and razor blade, and the tails were cut into 2-3 mm pieces using sterile surgical scissors. These pieces were then transferred into a 2 mL cryotube containing a collagenase D–pronase solution and left in a shaking incubator at 37^o^C for 90 minutes. Following incubation, the contents of the cryotubes were placed into a 70 µm cell strainer in a new 10-cm dish containing 10 mL of compete medium. Using the back end of a sterile 10 mL syringe plunger, the tissue was ground into the strainer for 5-10 minutes to release the cells into the media. The cell suspension was then collected into 15 mL conical tubes and centrifuged at 580 x g and 4^o^C for 5 minutes. The supernatant was removed and replaced with fresh complete medium, and the centrifugation was repeated for two additional rounds. After the final spin, the media was replaced with complete medium supplemented with 250 ng/mL of amphotericin B. The cell suspension was placed in a 10-cm dish and incubated in a 37^o^C humidified incubator at 3% O_2_ and 10% CO_2_ for two days before passage and continued culture in complete medium (DMEM, 10% FBS, 1% penicillin-streptomycin). This complete medium was used for the remainder of our experiments. For long term storage, cells were suspended in 5% DMSO in FBS, aliquoted into cryotubes, and placed in a slow cooling container in a -80^o^C freezer overnight. The following day, the cryotubes were moved out of the slow cooling container and placed into long term containers in the freezer. To thaw cells for continued culture, cryotubes containing frozen aliquots were swirled in a 37^o^C water bath until only a thin layer of ice remained in the vial. The contents of the cryotube were then immediate transferred into a flask containing pre-warmed complete medium and incubated overnight. The following day the cells were washed twice with PBS to remove any trace of DMSO and cultured in complete medium.

For experiments in wild-type *M. musculus*, *M. spretus*, and F1 hybrid cells we considered the culture from each individual animal to represent one biological replicate of the respective genotype. In all setups, we refer to the split cultures from a given biological replicate as technical replicates in a given assay. In experiments assessing cell growth and DNA damage during senescence in purebred *M. musculus* and *M. spretus* cells, we employed two such cultures of each genotype, which we refer to as biological replicates. In experiments of SABG and gene expression profiling during senescence, we employed three biological replicates of each genotype. Proteomic analysis was carried out in one biological replicate of each genotype. In experiments of SABG in purebred *M. domesticus* cells, we employed one biological replicate. Three biological replicates of F1 hybrid cells were used in our transcriptomic screen for senescence regulators. In experiments of SABG in F1 hybrid cells, one such biological replicate was used. In knockdown experiments we carried out infection of two biological replicates with each shRNA-bearing virus. Biological replicates from a given species were chosen at random for each experiment.

**Proteomic analysis of secreted proteins**

*Conditioned medium preparation.* For a given replicate culture, either before irradiation or 10 days after irradiation (see above), cells were washed three times with PBS and incubated with serum and phenol red free DMEM containing 1% pen-strep for 24 hours. The following day the conditioned medium was collected and passed through a 0.45 µm filter to remove cellular debris. The conditioned medium was placed in a -80^o^C freezer for storage before use as input into proteomic profiling (see below). For proteomic profiles of purebred cells, we carried out this procedure for three technical replicate cultures of one biological replicate per species.

*Sample concentration.* 30 mL of conditioned media for each replicate were concentrated to 400 µL with 15 mL 3 kDa filters (Millipore Sigma, Burlington, MA). Protein concentration was determined using the Bicinchoninic Acid (BCA) assay (Thermo Fisher Scientific, Waltham, MA).

*Protein digestion and desalting.* Aliquots of 200 µg protein lysates for each sample were brought to the same overall volume of 52 µL with water, reduced using 20 mM dithiothreitol in 50 mM triethylammonium bicarbonate buffer (TEAB) at 50^o^C for 10 min, cooled to room temperature (RT) and held at RT for 10 min, and alkylated using 40 mM iodoacetamide in 50 mM TEAB at RT in the dark for 30 min. Samples were acidified with 12% phosphoric acid to obtain a final concentration of 1.2% phosphoric acid. S-Trap buffer consisting of 90% methanol in 100 mM TEAB at pH ~7.1*,* was added and samples were loaded onto the S-Trap mini spin columns. The entire sample volume was spun through the S-Trap mini spin columns at 4,000 x g and RT, binding the proteins to the mini spin columns. Subsequently, S-Trap mini spin columns were washed twice with S-Trap buffer at 4,000 x g at RT and placed into clean elution tubes. Samples were incubated for one hour at 47^o^C with sequencing grade trypsin (Promega, San Luis Obispo, CA) dissolved in 50 mM TEAB at a 1:25 (w/w) enzyme:protein ratio. Afterwards, trypsin solution was added again at the same ratio, and proteins were digested overnight at 37^o^C.

Peptides were sequentially eluted from mini S-Trap spin columns with 50 mM TEAB, 0.5% formic acid (FA) in water, and 50% acetonitrile (ACN) in 0.5% FA. After centrifugal evaporation, samples were resuspended in 0.2% FA in water and desalted with Oasis 10 mg Sorbent Cartridges (Waters, Milford, MA). The desalted elutions were then subjected to an additional round of centrifugal evaporation and re-suspended in 0.2% FA in water at a final concentration of 1 µg/µL. Subsequently, 8 µL of each sample were diluted with 2% ACN in 0.1% FA to obtain a concentration of 400 ng/µL. Finally, 1 µL of indexed Retention Time Standard (iRT, Biognosys, Schlieren, Switzerland) was added to each sample, thus bringing up the total volume to 20 µL (Escher et al. 2012).

*Mass spectrometric analysis.* Reverse-phase HPLC-MS/MS analyses were performed on a Dionex UltiMate 3000 system coupled online to an Orbitrap Exploris 480 mass spectrometer (Thermo Fisher Scientific, Bremen, Germany). The solvent system consisted of 2% ACN, 0.1% FA in water (solvent A) and 80% ACN, 0.1% FA in ACN (solvent B). Digested peptides (400 ng) were loaded onto an Acclaim PepMap 100 C_18_ trap column (0.1 x 20 mm, 5 µm particle size; Thermo Fisher Scientific) over 5 min at 5 µL/min with 100% solvent A. Peptides (400 ng) were eluted on an Acclaim PepMap 100 C_18_ analytical column (75 µm x 50 cm, 3 µm particle size; Thermo Fisher Scientific) at 300 nL/min using the following gradient: linear from 2.5% to 24.5% of solvent B in 125 min, linear from 24.5% to 39.2% of solvent B in 40 min, up to 98% of solvent B in 1 min, and back to 2.5% of solvent B in 1 min. The column was re-equilibrated for 30 min with 2.5% of solvent B, and the total gradient length was 210 min. Each sample was acquired in data-independent acquisition (DIA) mode (Gillet et al. 2012; Bruderer et al. 2017), in triplicates. Full MS spectra were collected at 120,000 resolution (Automatic Gain Control (AGC) target: 3e6 ions, maximum injection time: 60 ms, 350-1,650 *m/z*), and MS2 spectra at 30,000 resolution (AGC target: 3e6 ions, maximum injection time: Auto, Normalized Collision Energy (NCE): 30, fixed first mass 200 *m/z*). The isolation scheme consisted of 26 variable windows covering the 350-1,650 *m/z* range with an overlap of 1 *m/z* (Collins et al. 2017).

*DIA data processing and statistical analysis.* DIA data was processed in Spectronaut (version 15.6.211220.50606) using directDIA. Data extraction parameters were set as dynamic and non-linear iRT calibration with precision iRT was selected. Data was searched against the *Mus musculus* reference proteome with 58,430 entries (UniProtKB-TrEMBL), accessed on 01/31/2018. Trypsin/P was set as the digestion enzyme and two missed cleavages were allowed. Cysteine carbamidomethylation was set as a fixed modification; methionine oxidation and protein N-terminus acetylation were set as dynamic modifications. Identification was performed using 1% precursor and protein q-value. Quantification was based on the peak areas of extracted ion chromatograms (XICs) of 3 – 6 MS2 fragment ions, specifically b- and y-ions, with local normalization and q-value sparse data filtering applied (Table S4). In addition, iRT profiling was selected. Senescence specific differential protein secretion analysis comparing samples derived from unirradiated and senescent *M. musculus* and *M. spretus* cells was performed using the proteomics data as input for a two-factor ANOVA with Benjamini-Hochberg multiple testing correction (Benjamini and Hochberg 1995). Only those proteins with a corrected *p*-value < 0.05 were considered significantly differentially secreted between the species during senescence.

**Cell proliferation and DNA damage assays**

For each of two biological replicates of purebred *M. musculus* (PWK) cells infected with lentivirus harboring the scrambled control and two of each *Usf2* knockdown, either before irradiation or 6 hours after irradiation (see above), we measured cell proliferation and DNA damage response as follows.

For a given replicate, DNA synthesis was measured via 5-ethynyl-2´-deoxyuridine (EdU) incorporation assays using the Invitrogen^TM^ Click-iT^TM^ Edu Alexa Fluor^TM^ 488 Flow Cytometry Assay Kit (cat. #C10420). Cells were treated with 5 µM EdU in complete medium and left in the incubator overnight. The following day cells were fixed, permeabilized, and treated with Alexa Fluor^TM^ 488 azide before running through a BD LSRFortessa^TM^ Cell Analyzer to identify the percentage of EdU positive cells.

For a given replicate, we carried out a comet assay to measure levels of DNA double stranded breaks for a given replicate culture as described (Olive and Banáth 2006). Briefly, slides scored with a diamond tipped scribe were dipped in 1% low melting point agarose and left at 4^o^C overnight. Cells of all genotypes tested were mixed into 1% low melting point agarose at 2 × 10^4^ cells/ml, spread over the prepared slides and left to solidify for 1 hour. Slides were then incubated at 37^o^C in 2% sarkosyl, 0.5 M NA_2_EDTA and 0.5 mg/mL proteinase K pH 8 lysis buffer overnight. The following day the slides were washed three times in 90 mM Tris, 90 mM boric acid, and 2 mM NA_2_EDTA pH 8.5 wash buffer, then subject to electrophoresis at 0.6 V/cm for 25 minutes in wash buffer. The slides were then washed three times in distilled water and placed in a staining solution containing 5 µg/mL of propidium iodide for 20 minutes. The slides were washed again once with distilled water and multiple representative images were taken of each sample using a Zeiss AxioObserver epi-fluorescent microscope. Images were processed via ZEN Digital Imaging for Light Microscopy (RRID:SCR_013672) and comet tail moments were analyzed via OpenComet (Gyori et al. 2014) in ImageJ (Schneider et al. 2012).

For H2AX assays, for a given replicate, cells were cultured in 8-chamber Nunc^TM^ Lab-Tek^TM^ II Chamber Slides^TM^ (Thermo Fisher cat. # 154453). The day before staining, all cells were seeded at 60-70% confluence in each chamber. Cells were fixed by incubating with 4% paraformaldehyde for 5 minutes at 4^o^C and permeabilized using 0.1% Triton X-100 for 15 minutes at room temperature. The cells were then blocked using 3% bovine serum albumin (BSA) in PBS for 45 minutes at room temperature, then incubated with 1 µg/mL of primary antibodies specific to phosphorylated (Ser 139) H2AX (cat. # sc-517348, Santa Cruz Biotechnology) in 3% BSA overnight at 4^o^C. The following day the cells were washed in PBS three times before incubating with 2 µg/mL of Alexa 488 secondary antibodies purchased from Invitrogen (cat. # A11001) for two hours at room temperature. Cells were washed three times with PBS then incubated with 0.5 µg/mL DAPI for 5 minutes at room temperature. The cells were washed once more with PBS before mounting for imaging. Multiple representative confocal images of each sample were taken using a Zeiss LSM 710 AxioObserver. Images were processed using ImageJ, taking the average background fluorescence across several nuclei in each field of vision and only counting foci with fluorescence intensities above the background threshold.

In addition to the above, we also carried out EdU incorporation assays on two biological replicates of wild-type primary fibroblasts of the *M. musculus* (PWK) background at five days after irradiation, and observed 0.5 and 1% EdU staining, respectively.

**References**

Benjamini Y, Hochberg Y. 1995. Controlling the False Discovery Rate: A Practical and Powerful Approach to Multiple Testing. J R Stat Soc Ser B Methodol. 57(1):289–300.

Bruderer R, Bernhardt OM, Gandhi T, Xuan Y, Sondermann J, Schmidt M, Gomez-Varela D, Reiter L. 2017. Optimization of Experimental Parameters in Data-Independent Mass Spectrometry Significantly Increases Depth and Reproducibility of Results. Mol Cell Proteomics MCP. 16(12):2296–2309. doi:10.1074/mcp.RA117.000314.

Collins BC, Hunter CL, Liu Y, Schilling B, Rosenberger G, Bader SL, Chan DW, Gibson BW, Gingras A-C, Held JM, et al. 2017. Multi-laboratory assessment of reproducibility, qualitative and quantitative performance of SWATH-mass spectrometry. Nat Commun. 8(1):291. doi:10.1038/s41467-017-00249-5.

Escher C, Reiter L, MacLean B, Ossola R, Herzog F, Chilton J, MacCoss MJ, Rinner O. 2012. Using iRT, a normalized retention time for more targeted measurement of peptides. Proteomics. 12(8):1111–1121. doi:10.1002/pmic.201100463.

Gillet LC, Navarro P, Tate S, Röst H, Selevsek N, Reiter L, Bonner R, Aebersold R. 2012. Targeted Data Extraction of the MS/MS Spectra Generated by Data-independent Acquisition: A New Concept for Consistent and Accurate Proteome Analysis*. Mol Cell Proteomics. 11(6):O111.016717. doi:10.1074/mcp.O111.016717.

Gyori BM, Venkatachalam G, Thiagarajan PS, Hsu D, Clement M-V. 2014. OpenComet: An automated tool for comet assay image analysis. Redox Biol. 2:457–465. doi:10.1016/j.redox.2013.12.020.

Khan M, Gasser S. 2016. Generating Primary Fibroblast Cultures from Mouse Ear and Tail Tissues. J Vis Exp JoVE.(107). doi:10.3791/53565. [accessed 2022 Mar 23]. https://www.ncbi.nlm.nih.gov/pmc/articles/PMC4781275/.

Olive PL, Banáth JP. 2006. The comet assay: a method to measure DNA damage in individual cells. Nat Protoc. 1(1):23–29. doi:10.1038/nprot.2006.5.

Schneider CA, Rasband WS, Eliceiri KW. 2012. NIH Image to ImageJ: 25 years of image analysis. Nat Methods. 9(7):671–675. doi:10.1038/nmeth.2089.
