# Supplementary material for: A natural variation-based screen in mouse cells reveals USF2 as a regulator of the DNA damage response and cellular senescence
Source: G3 (Bethesda). 2023 Apr 25;13(7):jkad091. doi: 10.1093/g3journal/jkad091 (PMC10320765; doi:10.1093/g3journal/jkad091)
Supplement: jkad091_Supplementary_Data [file jkad091_supplementary_data.zip › Supplemental_Table_Captions_G3-2023-404074.docx]

Supplemental table captions

**Table S1: RNA-seq gene expression profiling of resting and senescent purebred *M. musculus* and *M. spretus* primary cells.** Each row reports expression of the indicated gene in transcripts per million, as an average across biological replicates of primary fibroblasts from the indicated background cultured in the indicated condition. Columns report expression in purebred *M. musculus* (Mm) or *M. spretus* (Ms) primary fibroblasts, or expression from each allele (*M. musculus*, Mm; *M. spretus*, Ms) in primary fibroblasts from the interspecific F1 hybrid, in unirradiated control (CONT) or 10 days after irradiation (senescent, SEN).

**Table S2: Functional-genomic enrichment analysis of expression profiles of unirradiated purebred *M. musculus* and *M. spretus* primary cells.** Each row reports the results of a resampling test for enrichment of expression change between unirradiated primary fibroblasts from purebred *M. musculus* and *M. spretus* (see Table S1) for the indicated Gene Ontology biological process term. The second column represents the absolute value of the sum of the ratios of expression in *M. musculus* versus *M. spretus* for all the genes in the indicated term. The third column reports the number of genes with expression data in the term. The fourth column reports the raw *p*-value from resampling analysis, and the final column reports Benjamini-Hochberg corrected *p*-values. Only terms with significant enrichment (corrected *p* < 0.05) are shown.

**Table S3: Functional-genomic enrichment analysis of expression profiles of senescent purebred *M. musculus* and *M. spretus* primary cells.** Data are as in Table S2 except that *M. musculus* and *M. spretus* cells were analyzed 10 days after irradiation (see Table S1).

**Table S4: Proteomic profiling of conditioned medium from purebred *M. musculus* and *M. spretus* primary cells via mass spectrometry.** Columns report the abundance of the indicated protein via mass spectrometry, as an average over replicates and individual detected peptides, in conditioned medium from *M. musculus* (Mm) or *M. spretus* (Ms) cells, each as an unirradiated control (CONT) or 10 days after irradiation (senescent, SEN).

**Table S5: A screen correlating divergence in transcription factor binding sites and *cis-*regulatory expression during senescence.** Each row reports the results of a Fisher’s exact test relating, for the indicated transcription factor, two measures of variation between *M. musculus* and *M. spretus*: sequence variants at the factor’s experimentally determined binding sites upstream of genes, and, at these genes, senescence-specific expression differences between the two species’ alleles in fibroblasts of the F1 hybrid background. The second and third columns report nominal and Benjamini-Hochberg corrected *p*-values respectively.

**Table S6: Expression profiling of shRNA-expressing purebred *M. musculus* primary cells across a timecourse of irradiation and senescence.** Columns report expression of the indicated gene in transcripts per million, as an average across biological replicates, in purebred primary fibroblasts from *M. musculus* (PWK) subjected to either of two regimes. In one, cells were first infected with lentivirus expressing the indicated short hairpin RNA (shRNA) and then profiled before (CONT) or 6 hours (6HR) or 10 days (SEN.10d) after irradiation. In the other regime, cells were irradiated, then infected with lentivirus expressing the indicated shRNA, and then profiled 20 days later (SEN.20d). Usf2, *Usf2* targeting shRNA; SCR, scrambled control shRNA.

**Table S7: Functional-genomic enrichment analysis of purebred *M. musculus* primary cell transcriptomes upon *Usf2* knockdown and irradiation.** Data are as in Table S2 except that *M. musculus* (PWK) primary cells harboring short hairpin RNAs targeting *Usf2* or a scrambled control were treated with ionizing radiation, and profiled 6 hours afterward (see Table S6); for each gene we formulated the average expression measurement across replicates from each shRNA category in turn, and we then took the ratio between these averages for input into Gene Ontology enrichment tests.

**Table S8:** **Functional-genomic enrichment analysis of purebred *M. musculus* primary cell transcriptomes upon *Usf2* knockdown, irradiation, and senescence establishment.** Data are as in Table S7 except that cells were infected with lentivirus expressing the indicated short hairpin RNA (shRNA), treated with ionizing radiation, and profiled 10 days afterward (see Table S6).

**Table S9: Expression analysis of purebred *M. musculus* primary cells across a timecourse of irradiation and senescence.** Each row reports results from analysis of expression of one gene in *M. musculus* (PWK) primary cells harboring a scrambled short hairpin RNA, before and 6 hours, 10 days, and 20 days after irradiation (see Table S6). Second and third columns report the raw and Benjamini-Hochberg corrected *p*-values respectively from a multivariate ANOVA test for variation in expression across the timecourse. Only genes with significant differential expression across the timecourse (corrected *p* < 0.05) are shown.

**Table S10:** **Functional-genomic enrichment analysis of purebred *M. musculus* primary cell transcriptomes upon irradiation, senescence establishment, *Usf2* knockdown, and long-term senescence.** Data are as in Table S7 except that *M. musculus* (PWK) primary cells were irradiated, incubated for 10 days, infected with lentivirus harboring short hairpin RNAs targeting *Usf2* or a scrambled control, and allowed to maintain senescence for another 10 days (see Table S6); for each gene we formulated the average expression measurement across replicates from each shRNA category in turn, and we then took the ratio between these averages for input into Gene Ontology enrichment tests.

**Table S11: *In silico* regulatory network reconstruction from irradiation and senescence transcriptomes.** Shown are results from regulatory network inference using all shRNA treated transcriptomes in this study (see Table S6) as input into the MERLIN package. Each row reports results from a transcription factor inferred to be a downstream target of USF2. The second column reports the strength of the inferred regulation by USF2; negative values indicate repression. In the remaining columns, “Yes” indicates that one or more genes of the indicated GO term were inferred to be targets of the indicated factor.
